# Inferring networks from time series: A neural approach

**DOI:** 10.1093/pnasnexus/pgae063

**Published:** 2024-02-09

**Authors:** Thomas Gaskin, Grigorios A Pavliotis, Mark Girolami

**Affiliations:** Department of Applied Mathematics and Theoretical Physics, University of Cambridge, Cambridge CB3 0WA, UK; Department of Mathematics, Imperial College London, London SW7 2AZ, UK; Department of Mathematics, Imperial College London, London SW7 2AZ, UK; Department of Engineering, University of Cambridge, Cambridge CB2 1PZ, UK; The Alan Turing Institute, London NW1 2DB, UK

**Keywords:** network inference, neural differential equations, model calibration, power grids

## Abstract

Network structures underlie the dynamics of many complex phenomena, from gene regulation and foodwebs to power grids and social media. Yet, as they often cannot be observed directly, their connectivities must be inferred from observations of the dynamics to which they give rise. In this work, we present a powerful computational method to infer large network adjacency matrices from time series data using a neural network, in order to provide uncertainty quantification on the prediction in a manner that reflects both the degree to which the inference problem is underdetermined as well as the noise on the data. This is a feature that other approaches have hitherto been lacking. We demonstrate our method’s capabilities by inferring line failure locations in the British power grid from its response to a power cut, providing probability densities on each edge and allowing the use of hypothesis testing to make meaningful probabilistic statements about the location of the cut. Our method is significantly more accurate than both Markov-chain Monte Carlo sampling and least squares regression on noisy data and when the problem is underdetermined, while naturally extending to the case of nonlinear dynamics, which we demonstrate by learning an entire cost matrix for a nonlinear model of economic activity in Greater London. Not having been specifically engineered for network inference, this method in fact represents a general parameter estimation scheme that is applicable to any high-dimensional parameter space.

**Significance Statement**

In this work, we learn static network structures from time series, an important problem across the quantitative sciences, where networks can appear either as physical links or abstract connections, but in many cases will only be indirectly observable through their dynamics. We show our method to be more accurate, computationally efficient, and versatile both than Markov-chain Monte Carlo sampling and regression. Additionally, it provides meaningful uncertainty quantification on the network prediction, which is both novel and key, as it allows estimating the range of networks compatible with the observation data, which may not fully determine the inference problem. Our method thus lets researchers make probabilistic statements about the connectivity matrices underlying the dynamics they observe.

## Introduction

Networks are important objects of study across the scientific disciplines. They materialize as physical connections in the natural world, for instance as the mycorrhizal connections between fungi and root networks that transport nutrients and warning signals between plants (1, 2), human traffic networks (3, 4), or electricity grids (5, 6). However, they also appear as abstract, nonphysical entities, such as when describing biological interaction networks and food webs (7–9), gene or protein networks (10–13), economic cost relations (14, 15), or social links between people along which information (and misinformation) can flow (16–18). In all examples, though the links constituting the network may not be tangible, the mathematical description is the same. In this work, we are concerned with inferring the structure of a static network from observations of dynamics on it. The problem is of great scientific bearing: for instance, one may wish to understand the topology of an online social network from observing how information is passed through it, and some work has been done on this question (19–21). Another important application is inferring the connectivity of neurons in the brain by observing their responses to external stimuli (22, 23). In an entirely different setting, networks crop up in statistics in the form of conditional independence graphs, describing dependencies between different variables, which again are to be inferred from data (24, 25).

Our approach allows inferring network connectivities from time series data with uncertainty quantification. Uncertainty quantification for network inference is important for two reasons: first, the observations will often be noisy, and one would like the uncertainty on the data to translate to the uncertainty on the predicted network. Second, however, completely inferring large networks requires equally large amounts of data—typically at least N−1 equations per node, *N* being the number of nodes—and these observations must furthermore be linearly independent. Data of such quality and quantity will often not be available, leading to an underdetermined inference problem. The uncertainty on the predicted network should thus also reflect (at least to a certain degree) the “nonconvexity” of the loss function, i.e. how many networks are compatible with the observed data. To the best of our knowledge, no current network inference method is able to provide this information.

Network inference can be performed using ordinary least squares (OLS) regression (6, 26), but this is confined to the case where the dynamics are linear in the adjacency matrix. An alternative are sampling-based methods that generalize to the nonlinear case (27–29), but these tend to struggle in very high-dimensional settings and can be computationally expensive. Efficient inference methods for large networks have been developed for cascading dynamics (19–21), but these are highly specialized to a particular type of observation data and give no uncertainty quantification on the network prediction. Our method avoids these limitations. Its use of neural networks is motivated by their recent and successful application to low-dimensional parameter calibration problems (30, 31), both on synthetic and real data, as well as by their conceptual proximity to Bayesian inference, e.g. through neural network Gaussian processes or Bayesian neural networks (32–37). Our method’s underlying approach ties into this connection, and in fact, since it has not been specifically engineered to fit the network case, constitutes a general and versatile parameter estimation method.

### Method description

We apply the method proposed in Ref. (31) to the network case. The approach consists of training a neural network to find a graph adjacency matrix $\hat{A}\in R^{N\times N}$ that, when inserted into the model equations, reproduces the observed time series $T=(x_{1},\ldots ,x_{L})$. A neural network is a function $u_{\theta }\;:\;R^{N\times q}\to R^{p}$, where q≥1 represents the number of time series steps that are passed as input. Its output is the (vectorized) estimated adjacency matrix $\hat{A}$, which is used to run a numerical solver for B iterations (B is the batch size) to produce an estimated time series $\hat{T}(\hat{A})=({\hat{x}}_{i},\ldots ,{\hat{x}}_{i+B})$. This in turn is used to train the internal parameters θ of the neural net (the weights and biases) via a loss function $J(\hat{A}\;|\;T)$. The likelihood of any sampled estimate is simply proportional to


(1) $$p\left(\hat{A}\;|\;T\right)\propto e^{-J}$$

and by Bayes’ rule, the posterior density is then


(2) $$\pi \left(\hat{A}\;|\;T\right)=p\left(\hat{A}\;|\;T\right)\times \pi^{0}(\hat{A}),$$

with $\pi^{0}$ the prior density (38). As $\hat{A}=\hat{A}(\theta )$, we may calculate the gradient $\nabla_{\theta }J$ and use it to optimize the internal parameters of the neural net using a backpropagation method of choice; popular choices include stochastic gradient descent, Nesterov schemes, or the Adam optimizer (39). Calculating $\nabla_{\theta }J$ thus requires differentiating the predicted time series $\hat{T}$, and thereby the system equations, with respect to $\hat{A}$. In other words, the loss function contains knowledge of the dynamics of the model. Finally, the true data are once again input to the neural net to produce a new parameter estimate $\hat{A}$, and the cycle starts afresh. A single pass over the entire dataset is called an epoch.

Using a neural net allows us to exploit the fact that, as the net trains, it traverses the parameter space, calculating a loss at each point. Unlike Monte Carlo sampling, the posterior density is not constructed from the frequency with which each point is sampled, but rather calculated directly from the loss value at each sample point. This entirely eliminates the need for rejection sampling or a burn-in: at each point, the true value of the likelihood is obtained, and sampling a single point multiple times provides no additional information, leading to a significant improvement in computational speed. Since the stochastic sampling process is entirely gradient-driven, the regions of high probability are typically found much more rapidly than with a random sampler, leading to a high sample density around the modes of the target distribution. We thus track the neural network’s path through the parameter space and gather the loss values along the way. Multiple training runs can be performed in parallel, and each chain terminated once it reaches a stable minimum, increasing the sampling density on the domain, and ensuring convergence to the posterior distribution in the limit of infinitely many chains.

We begin this article with two application studies: first, we infer locations of a line failure in the British power grid from observations of the network response to the cut; and second, we infer economic cost relations between retail centers in Greater London. Thereafter, we conduct a comparative analysis of our method’s performance, before finally demonstrating the connection between the uncertainty on the neural net prediction and the uncertainty of the inference problem.

## Inferring line failures in the British power grid

Power grids can be modeled as networks of coupled oscillators using the Kuramoto model of synchronized oscillation (40–44). Each node i in the network either produces or consumes electrical power $P_{i}$ while oscillating at the grid reference frequency Ω. The nodes are connected through a weighted undirected network $A=(a_{ij})$, where the link weights $a_{ij}∼Y_{ij}U_{ij}^{2}$ are obtained from the electrical admittances $Y_{ij}$ and the voltages $U_{ij}$ of the lines. The network coupling allows the phases $\varphi_{i}(t)$ of the nodes to synchronize according to the differential equation (43)


(3) $$\alpha \frac{d^{2}\varphi_{i}}{dt^{2}}+\beta \frac{d\varphi_{i}}{dt}=P_{i}+\kappa \sum_{j}a_{ij}\sin\;(\varphi_{j}-\varphi_{i}),$$

where α, β, and κ are the inertia, friction, and coupling coefficients, respectively. A requirement for dynamical stability of the grid is that $\sum_{i}P_{i}=0$, i.e. that as much power is put into the grid as is taken out through consumption and energy dissipation (42).

**Fig. 1.**
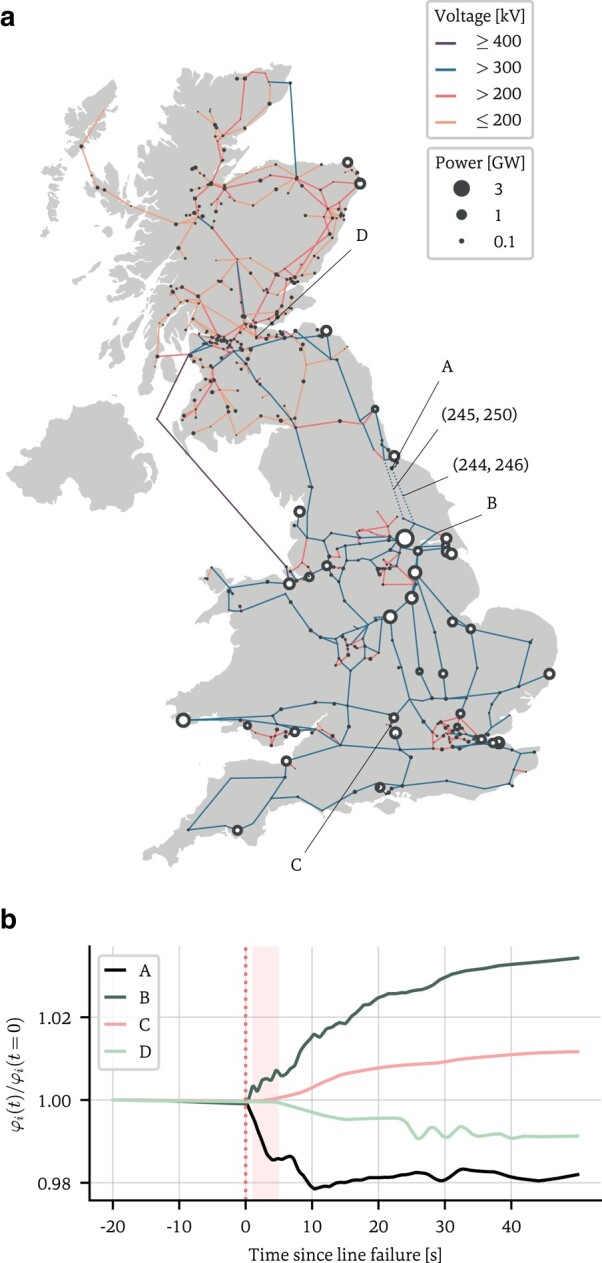
a) Approximate high-voltage electricity transmission grid of Great Britain. Six hundred and thirty accurately placed nodes, representing power stations, substations, and transmission line intersections, and their connectivity as of January 2023 are shown (46–48). Colors indicate the operating voltage of the lines. The size of the nodes indicate their power generation or consumption capacity (absolute values shown). White ringed nodes indicate the 38 nodes that are real power stations with capacities over 400 MW (45), with all other nodes assigned a random capacity in [−200,+200]. The two dotted edges in the northeast of England are the edges affected by a simulated power cut, labeled by the indices of their start and end vertices. b) The network response to the simulated power line failure, measured at four different nodes in the network (marked A–D). The equation parameters were tuned to ensure phase-locking of the oscillators ( α=1, β=0.2, κ=60). Nodes closer to the location of the line cut (A and B) show a stronger and more immediate response than nodes further away (C and D). The shaded area indicates the 4-second window we use to infer the line location. Background image: (49).

A power line failure causes the network to redistribute the power loads, causing an adjustment cascade to ripple through the network until equilibrium is restored (5). In this work, we recover the location of a line failure in the British power grid from observing these response dynamics. Figure 1a shows the high-voltage transmission grid of Great Britain as of January 2023, totalling 630 nodes (representing power stations, substations, and transformers) and 763 edges with their operating voltages. Of the roughly 1,300 power stations dotted around the island, we include those 38 with installed capacities of at least 400 MW that are directly connected to the national grid (45); following Refs. (5, 42), we give all other nodes a random value $P_{i}∼U[-200,+200]$ such that $\sum_{i}P_{i}=0$.

We simulate a power cut in the northeast of England by iterating the Kuramoto dynamics until the system reaches a steady state of equilibrium (defined as $|{\dot{\varphi }}_{i}|/\varphi_{i}\leq 0.01\;\forall i$) and then removing two links and recording the network response (Fig. 1b). From the response, we can infer the adjacency matrix of the perturbed network $\tilde{A}$ (with missing links) and, by comparing with the unperturbed network $A^{0}$ (without missing links), the line failure locations.

We let a neural network output a (vectorized) adjacency matrix $\hat{A}$ and use this estimated adjacency matrix to run the differential equation [3], which will produce an estimate $\hat{T}$ of the observed time series of phases T. A hyperparameter sweep on synthetic data showed that using a deep neural network with 5 layers, 20 nodes per layer, and no bias yields optimal results (see Figs. S2–S4). We use the hyperbolic tangent as an activation function on each layer except the last, where we use the “hard sigmoid” (50, 51)


$$\sigma (x)=\left\{ \begin{array}{cc} 0, & x\leq -3,\\ 1, & x\geq +3,\\ x/6+1/2, & \mathrm{else}, \end{array}\right.$$

which allows neural net output components to actually become zero, and not just asymptotically close, thereby ensuring sparsity of the adjacency matrix—a reasonable assumption given that the power grid is far from fully connected. We use the Adam optimizer (39) with a learning rate of 0.002 for the gradient descent step. Since the neural network outputs are in [0,1], we scale the network weights $a_{ij}\to \lambda a_{ij}$ such that $a_{ij}\in [0,1]$, and absorb the scaling constant λ into the coupling constant κ; see Supplementary material for details on the calculations.

We use the following loss function to train the internal weights θ of the neural network such that it will output an adjacency matrix that reproduces the observed data:


$$\begin{array}{rl} J\left(\hat{A}\;|\;T\right) & =\|\hat{T}(\hat{A})-T{\|}_{2}^{2}+\|\hat{A}-{\hat{A}}^{⊤}{\|}_{2}^{2}+\mathrm{tr}(\hat{A})\\ & \;+\nu \|\hat{A}-A^{0}{\|}_{2}^{2}. \end{array}$$

**Fig. 2.**
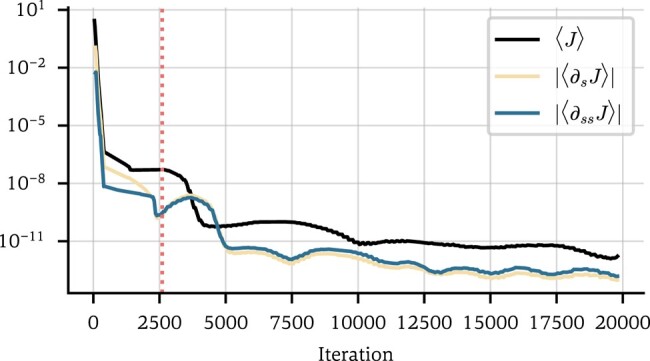
The total loss J and its derivatives with respect to the iteration count $\partial_{s}J$ and $\partial_{ss}J$, averaged over a window of 20 iterations (absolute values shown). The dotted line indicates the value at which ν is set to 0.

The first summand is the data-model mismatch, the second penalizes asymmetry to enforce undirectedness of the network, and the third sets the diagonal to zero (which cannot be inferred from the data, since all terms $\sin\;(\theta_{j}-\theta_{i})=0$ for i=j). ν=ν(s) is a function of the iteration count s designed to let the neural network search for $\tilde{A}$ in the vicinity of $A^{0}$, since we can assume a priori that the two will be similar in most entries. To this end, we set ν=10 while the loss function has not yet reached a stable minimum, quantified by $|\langle \partial_{s}J\rangle |>10^{-10}$ and $|\langle \partial_{ss}J\rangle |>10^{-10}$, and ν=0 thereafter. Here, ⟨⋅⟩ is a rolling average over a window of 20 iterations, see Fig. 2. In other words, we push the neural network toward a stable minimum in the neighborhood of $A^{0}$ and, once the loss stabilizes, permanently set ν=0.

In theory L=N−1, observations are needed to completely infer the network, though symmetries in the data usually mean L>N is required in practice (52). In this experiment, we purposefully underdetermine the problem by only using L<N−1 steps; additionally, we train the network on data recorded 1 simulated second after the power cut, where many nodes will still be close to equilibrium. Although the neural network may be unable to completely infer the network, it can nevertheless produce a joint distribution on the network edge weights $p(\hat{A}\;|\;T)$, recorded during the training, that allows us to perform hypothesis testing on the line failure location. The marginal likelihood on each network edge ${\hat{a}}_{ij}$ is given by


(4) $$\rho ({\hat{a}}_{ij}\;|\;T)\;=\int p\left(\hat{A}\;|\;T\right)d{\hat{A}}_{-ij}\times \pi^{0}({\hat{a}}_{ij}),$$

where the −ij subscript indicates we are omitting the ijth component of $\hat{A}$ in the integration. We assume uniform priors $\pi^{0}$ on each edge. In high dimensions, calculating the joint of all network edge weights can become computationally infeasible, but we can circumvent this by instead considering the two-dimensional joint density of the edge weight under consideration and the likelihood, $p({\hat{a}}_{ij},e^{-J})$, and then integrating over the likelihood,


(5) $$\rho ({\hat{a}}_{ij}\;|\;T)\;=\int p\left({\hat{a}}_{ij},e^{-J}\right)d(e^{-J}).$$

**Fig. 3.**
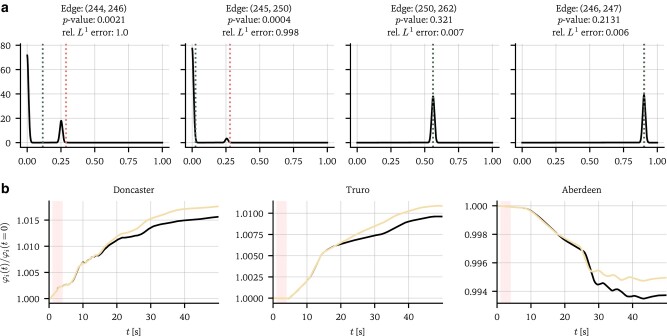
Estimating the line failure location. a) The densities on the four edges with the highest relative prediction error $|{\tilde{a}}_{ij}-a_{ij}^{0}|/a_{ij}^{0}$ and their respective *p*-values for measuring the unperturbed value $a_{ij}^{0}$ (${\tilde{a}}_{ij}$ is the prediction mode). Red dotted lines indicate the values of the unperturbed network, green lines the expectation values of the distributions. The marginals are smoothed using a Gaussian kernel. We use a training set of length L=400 steps, and the batch size is B=2. CPU runtime: 24 min. b) True (black) and predicted network responses at three different locations in the network. The responses are each normalized to the value at t=0. The shaded area represents the 400 time steps used to train the model. While the model is able to perfectly fit the response within the training range, it is not able to learn the full network from insufficient data, causing the time series to diverge for larger t.

We show the results in Fig. 3. Given the marginal distributions $\rho ({\hat{a}}_{ij}\;|\;T)$ with modes ${\tilde{a}}_{ij}$, we plot the densities on the four network edges with the highest relative prediction error $|{\tilde{a}}_{ij}-a_{ij}^{0}|/a_{ij}^{0}$. The advantage of obtaining uncertainty quantification on the network is now immediately clear: even in the underdetermined case, we are able to make meaningful statistical statements about the line failure location. We see that the missing edges consistently have the highest relative prediction errors, and that the *p*-values for measuring the unperturbed value $a_{ij}^{0}$ under the null ${\hat{a}}_{ij}$ are 0.2% and 0.04%, respectively, while being statistically insignificant for all other edges. It is interesting to note that the other candidate locations are also within the vicinity of the line failure, though their predicted values are much closer to the unperturbed value. In Fig. 3b, we see that the predicted network reproduces the response dynamics for the range covered by the training data when inserted into Eq. 3, but, since the problem was purposefully underdetermined, the errors in the prediction $\hat{A}$ cause the predicted and true time series to diverge for larger t. Densities on all 200,000 potential edges were obtained in about 20 min on a regular laptop CPU.

## Inferring economic cost networks from noisy data

**Fig. 4.**
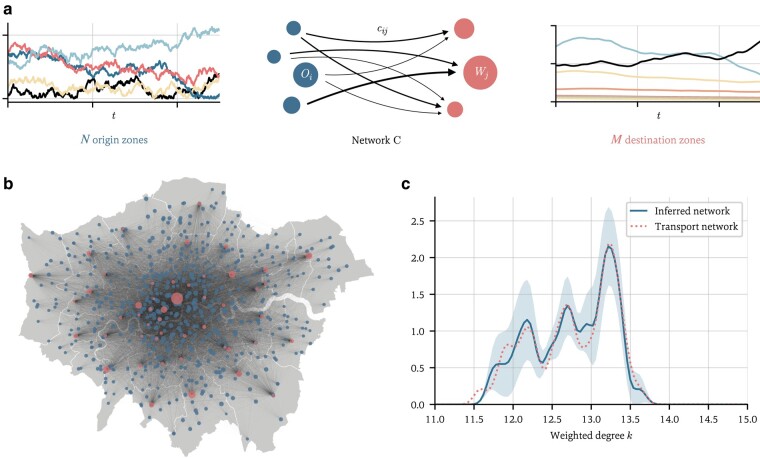
Inferring economic cost networks. a) In the model, N origin zones (red) are connected to M destination zones (blue) through a weighted directed network. Economic demand flows from the origin zones to the destination zones, which supply the demand. We model the origin zones $O_{i}(t)$ as a Wiener process with diffusion coefficient $\sigma_{O}=0.1$. The resulting cumulative demand at destination zone j is given by $W_{j}$. Note that the origin zone sizes fluctuate more rapidly than the destination zones, since there is a delay in the destination zones’ response to changing consumer patterns, controlled by the parameter ϵ. We use the parameters as estimated in Ref. (31), α=0.92, β=0.54, κ=8.3, and set ϵ=2. b) The initial origin and destination zone sizes, given by the total household income of the N=629 wards in London (blue nodes) and the retail floor space of M=49 major centers (red nodes) (53, 54). The network is given by travel times as detailed in the text. Background map: Ref. (55). c) Predicted degree distribution (sold line) of the inferred network, for a high noise level of σ=0.14, and 1 SD (shaded area), and the true distribution (dotted line). CPU runtime: 3 min 41 s.

In the previous example, the underlying network was a physical entity, but in many cases networks model abstract connections. We therefore now consider a commonly used economic model of the coupling of supply and demand (14, 15, 56) and a dataset of economic activity across Greater London. The goal is to learn the entire coupling network, not just to infer the (non)existence of individual edges. In the model, N origin zones of sizes $O_{i}$, representing economic demand, are coupled to M destination zones of sizes $W_{j}$, modeling the supply side, through a network whose weights quantify the convenience with which demand from zone i can be supplied from zone j: the higher the weight, the more demand flows through that edge (see Fig. 4a). Such a model is applicable e.g. to an urban setting (14), the origin zones representing residential areas, the destination zones e.g. commercial centers, and the weights quantifying the connectivity between the two (transport times, distances, etc.). The resulting cumulative demand at destination zone j depends both on the current size $W_{j}(t)$ of the destination zone and the network weights $c_{ij}$:


(6) $$D_{j}=\sum_{i=1}^{N}\frac{W_{j}{\left(t\right)}^{\alpha }c_{ij}^{\beta }}{\sum_{k=1}^{M}W_{k}{\left(t\right)}^{\alpha }c_{ik}^{\beta }}O_{i}(t).$$

The sizes $W_{j}$ are governed by a system of M coupled logistic Stratonovich stochastic differential equations


(7) $$dW_{j}=\epsilon W_{j}(D_{j}-\kappa W_{j})\;dt+\sigma W_{j}\circ d\xi_{j},$$

with given initial conditions $W_{j}(0)$, see Fig. 4a. α, β, κ, and ϵ are scalar parameters. Our goal is to infer the cost matrix $C=(c_{ij})$ from observations of the time series O(t) and W(t). The model includes multiplicative noise with strength σ≥0, where the $\xi_{j}$ are independent white noise processes and ○ signifies Stratonovich integration (57). Crucially, the model depends nonlinearly on C.

We apply this model to a previously studied dataset of economic activity in Greater London (15, 31). We use the ward-level household income from N=625 wards for 2015 (54) and the retail floor space of the M=49 largest commercial centers in London (53) as the initial origin zone and destination zone sizes, respectively, i.e. O(0) and W(0), and from this generate a synthetic time series using the parameters estimated in Ref. (31) for a high noise level of σ=0.14. For the network C, we use the Google Distance Matrix API^a^ to extract the shortest travel time $d_{ij}$ between nodes, using either public transport or driving. The network weights are derived in Ref. (58) as


$$c_{ij}=e^{-d_{ij}/\tau },$$

where the scale factor $\tau =\max_{i,j}d_{ij}$ ensures a unitless exponent.

We generate a synthetic time series of 10,000 time steps, from which we subsample 2,500 2-step windows, giving a total training set size of L=5,000 time steps. This is to ensure we sample a sufficiently broad spectrum of the system’s dynamics, thereby fully determining the inference problem and isolating the effect of the training noise. A hyperparameter sweep on synthetic data showed that using a neural network with 2 layers, 20 nodes per layer, and no bias yields optimal results. We use the hyperbolic tangent as the activation function on all layers except the last, where we use the standard sigmoid function (since the network is complete, there is no need to use the hard sigmoid as all edge weights are nonzero). To train the neural network, we use the simple loss function


$$J=\|\hat{T}(\hat{A})-T{\|}_{2}^{2},$$

where $\hat{T}$ and T are the predicted and true time series of destination zone sizes. Since the dynamics are invariant under scaling of the cost matrix C→λC, we normalize the row sums of the predicted and true networks, $\sum_{j}c_{ij}=1$.

Figure 4c shows the inferred distribution ρ(k) of the (weighted) origin zone node degrees $k_{j}=\sum_{i}c_{ij}$. The solid line is the maximum likelihood prediction, and the dotted line the true distribution. Even with a high level of noise, the model manages to accurately predict the underlying connectivity matrix, comprising over 30,000 weights, in under 5 min on a regular laptop CPU. Uncertainty on P(k) is given by the standard deviation,


(8) $$E_{\hat{T}}{\left[P\left(k\;|\;\hat{T}\right)-\hat{P}(k)\right]}^{2},$$

where $\hat{P}$ is the maximum likelihood estimator. As we will discuss in the last section, this method meaningfully captures the uncertainty due to the noise in the data and the degree to which the problem is underdetermined.

## Comparative performance analysis

We now analyze our method’s performance, both in terms of prediction quality and computational speed, by comparing it to a Markov-chain Monte Carlo (MCMC) approach as well as a classical regression method, presented e.g. in Refs. (6, 59). As mentioned in the Introduction section, computationally efficient network learning methods have been developed for specific data structures; however, we compare our approach with MCMC and OLS since both are general in the types of data to which they are applicable.

Consider noisy Kuramoto dynamics,


(9) $$\alpha \frac{d^{2}\varphi_{i}}{dt^{2}}+\frac{d\varphi_{i}}{dt}-\omega_{i}=\sum_{j}a_{ij}\sin\;(\varphi_{j}-\varphi_{i})+\xi_{i},$$

with $\xi_{i}$ independent white noise processes with strength σ and $\omega_{i}$ the eigenfrequencies of the nodes. Given L observations of each node’s dynamics, we can gather the left-hand side into a single vector $X_{i}\in R^{1\times L}$ for each node, and obtain N equations


(10) $$X_{i}=A_{i}\times G_{i}+\xi_{i},\;i=1,\ldots ,N,$$

with $A_{i}\in R^{1\times N}$ the ith row of the adjacency matrix A and $G_{i}\in R^{N\times L}$ the L observations of the interaction terms $\sin\;(\varphi_{j}-\varphi_{i}),$  j=1,…,N. From this, we can then naturally estimate the ith row of A using OLS:


(11) $${\hat{A}}_{i}=\underset{\gamma \in R^{1\times N}}{\mathrm{argmin}}\;\|X_{i}-\gamma G_{i}{\|}_{2}^{2}=X_{i}G_{i}^{⊤}{\left(G_{i}G_{i}^{⊤}\right)}^{-1}.$$

Given sufficiently many linearly independent observations, the Gram matrices $G_{i}G_{i}^{⊤}$ will all be invertible; in the underdetermined case, a pseudoinverse can be used to approximate their inverses. As before, the diagonal of $\hat{A}$ is manually set to 0.

In addition to regression, we also compare our method to a preconditioned Metropolis-adjusted Langevin (MALA) sampling scheme (27–29, 60), which constructs a Markov chain of sampled adjacency matrices $\hat{A}$ by drawing proposals from the normal distribution


(12) $${\hat{A}}^{i+1}∼N\left({\hat{A}}^{i}+\frac{\tau }{2}\lambda^{-1}P\nabla J\left({\hat{A}}^{i}\;|\;T\right),\tau \lambda^{-1}P\right).$$

Here, τ>0 is the integration step size, $P\in R^{N^{2}\times N^{2}}$ is a preconditioner (note that we are reshaping $\hat{A}$ into an $N^{2}$-dimensional vector), and $\lambda =\mathrm{tr}(P)/N^{2}$ is its average eigenvalue. Each proposal is accepted with probability


(13) $$\eta =\min\left[1,\frac{\exp\;\left(-J\left({\hat{A}}^{i+1}\right)\right)q\left({\hat{A}}^{i+1}\;|\;{\hat{A}}^{i}\right)}{\exp\;\left(-J\left({\hat{A}}^{i}\right)\right)q\left({\hat{A}}^{i}\;|\;{\hat{A}}^{i+1}\right)}\right],$$

with the transition probability


(14) $$q\left({\hat{A}}^{i+1}\;|\;{\hat{A}}^{i}\right)\propto \exp\;\left(-\frac{1}{4\tau }\|{\hat{A}}^{i+1}-{\hat{A}}^{i}-\tau \nabla \log\;\pi ({\hat{A}}^{I}){\|}_{2}^{2}\right).$$

We tune τ so that the acceptance ratio η converges to the optimum value of 0.57 (61).

We set the preconditioner P to be the inverse Fisher information covariance matrix


(15) $$P^{-1}=E_{\hat{A}}\left[\nabla J({\hat{A}}^{i})\nabla J({\hat{A}}^{i}{)}^{⊤}\right],$$

which has been shown to optimize the expected squared jump distance (29). The expectation value is calculated empirically over all samples drawn using the efficient algorithm given in Ref. (29). In all experiments, we employ a “warm start” by initializing the sampler close to the minimum of the problem. We found this to be necessary in such high dimensions (between 256 and 490,000) to produce decent results. Unlike the MCMC sampler, the neural network is initialized randomly.

**Fig. 5.**
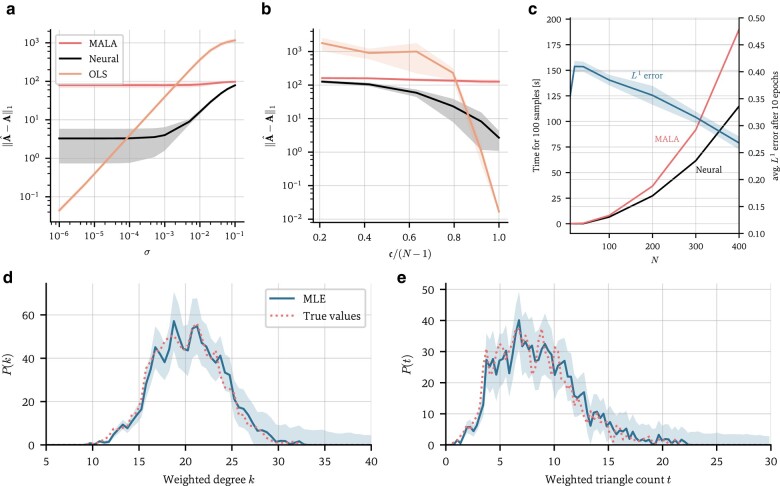
Computational performance analysis. a) $L^{1}$ prediction error (Eq. 16) of the neural scheme, the preconditioned Metropolis-adjusted Langevin sampler, and OLS regression as a function of the noise variance σ on the training data. For very high noise levels, the training data are essentially pure noise, and the prediction errors begin to plateau. First-order Kuramoto dynamics are used (α=0), though these results also hold for second-order dynamics (cf. Fig. S6). Enough data are used to ensure full invertibility of the Gram matrix (c=1). b) The $L^{1}$ accuracy as a function of the convexity c of the loss function (Eq. 17). c) Compute times for 10 epochs, or 100 samples, of the neural scheme and the preconditioned Metropolis-adjusted Langevin sampler, averaged over 10 runs. The shaded areas show one standard deviation. On the right axis, the average $L^{1}$ prediction error of the neural scheme $\frac{1}{N}\|\hat{A}-A{\|}_{1}$ after 10 epochs is shown, which remains fairly constant as a function of N, showing that the number of gradient descent steps required to achieve a given average prediction error does not depend on N. d) Predicted degree distribution and e) triangle distribution of an inferred network with N=1,000 nodes, trained on first-order noisy Kuramoto data (σ=0.001). The blue shaded areas indicate one standard deviation, and the dotted lines are the true distributions. CPU runtime: 1 h 3 min.

Figure 5a and b shows our method’s prediction accuracy alongside that of OLS regression and preconditioned MALA on synthetic Kuramoto data; the accuracy here is defined as the $L^{1}$ error


(16) $$\|\hat{A}-A{\|}_{1}=\sum_{i,j}|{\hat{a}}_{ij}-a_{ij}|,$$

where $\hat{A}$ is the mode of the posterior. In Fig. 5a, the accuracy is shown as a function of the noise σ on the training data. We generate enough data to ensure the likelihood function is unimodal. For the practically noiseless case of $\sigma <10^{-5}$, the regression scheme on average outperforms the neural approach; however, even for very low noise levels $\sigma \geq 10^{-5}$ and above, the neural approach proves far more robust, outperforming OLS by up to one order of magnitude and maintaining its prediction performance up to low noise levels of $\sigma \leq 10^{-3}$. Meanwhile, we find that in the low- to mid-level noise regime, the neural scheme approximates the mode of the distribution by between 1 and 2 orders of magnitude more accurately than the Langevin sampler. For high levels of noise ($\sigma >10^{-2}$), the performances of the neural and MALA schemes converge. These results hold both for first-order (α=0) and second-order Kuramoto dynamics [3]; in the second-order case, the neural method begins outperforming OLS at even lower levels of σ than in the first-order case, though the improvement is not as significant (cf. Fig. S6).

In Fig. 5b, we show the accuracy as a function of the convexity of the loss function. In general, it is hard to quantify the convexity of J, since we do not know how many networks fit the equation at hand. However, when the dynamics are linear in the adjacency matrix A, we can do so using the Gram matrices of the observations of each node i, $G_{i}G_{i}^{⊤}$, where we quantify the (non)convexity of the problem by the minimum rank of all the Gram matrices,


(17) $$c\;:=\min_{i}\mathrm{rk}\left(G_{i}G_{i}^{⊤}\right).$$

The problem is fully determined if c=N−1 and all Gram matrices are invertible. As shown, regression is again more accurate when the problem is close to fully determined; however, as c decreases, the accuracy quickly drops, with the neural scheme proving up to an order of magnitude more accurate. Meanwhile, the MCMC scheme is consistently outperformed by the neural scheme, though it too eclipses regression for c<0.75. In summary, regression is only viable for the virtually noiseless and fully determined case, while the neural scheme maintains good prediction performance even in the noisy and highly underdetermined case (see also Fig. 5d and e).

In Fig. 5c, we show compute times to obtain 100 samples for both the neural and MALA schemes. The complexity of the neural scheme is $O(n_{E}\times LN^{2})$, with $n_{E}$ the number of training epochs. This is because each epoch of the model equation requires $O(LN^{2})$ operations for the vector–matrix multiplication in Eq. 11, and $O(LN^{2}/B)$ for the stochastic gradient descent update, where we are holding L/B constant to ensure comparability. As is visible, the average $L^{1}$ error per edge weight remains constant over N, showing that the number of epochs required to achieve a given node-averaged prediction accuracy is independent of N. The preconditioned MALA scheme is considerably slower, due to the computational cost of calculating the preconditioner and the Metropolis–Hastings rejection step.

Lastly, Figs. 5d and e show the estimated weighted degree and triangle distributions of a large graph with 1,000 nodes, or 1 million edge weights to be estimated, for noisy training data. The number of weighted, undirected triangles on each node i is given by $\frac{1}{2}\sum_{\;jk}a_{ij}a_{\;jk}a_{ki}$. The model robustly finds the true adjacency matrix, and we again quantify uncertainty on the prediction using the standard deviation (Eq. 8). Estimating a network with 1,000 nodes on a standard laptop CPU took about 1 h, which reduces to 6 min when using a GPU. Most high-performance network inference techniques demonstrate their viability on graphs with at most this number of nodes, e.g. ConNIe (19) and NetINF (21). In Ref. (19), the authors state that graphs with 1,000 nodes can typically be inferred from cascade data in under 10 min on a standard laptop. Similarly, the authors of NetINF (21) state that it can infer a network with 1,000 nodes in a matter of minutes, though this algorithm does not infer edge weights, only the existence of edges, and neither technique provides uncertainty quantification.

## Quantifying uncertainty

**Fig. 6.**
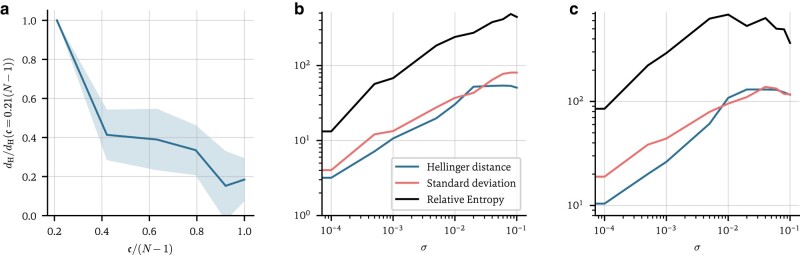
Quantifying the two types of uncertainty: a) Hellinger error (Eq. 18) on the degree distribution P(k) as a function of c (Eq. 17) in the noiseless case. The error is normalized to the value at c=0.21(N−1). As c increases, the error on the prediction decreases almost linearly. We run the model from 10 different initializations and average over each (shaded area: SD). b and c) Prediction uncertainty due to noise in the data. The expected Hellinger error (Eq. 18) and expected relative entropy (Eq. 19) to the maximum likelihood estimate, as well as the total SD s (Eq. 20) for the degree distribution P(k) and triangle distribution P(t) as a function of the noise σ on the data are shown. Each line is an average over 10 different initializations. In all cases, training was conducted on synthetic, first-order Kuramoto data (Eq. 9, with α=0).

There are two sources of uncertainty when inferring adjacency matrices: the nonconvexity of the loss function J and the noise σ on the data. In Fig. 6a, we show the expected Hellinger error


(18) $$\frac{1}{2}E_{\hat{T}}\int {\left[\sqrt{P\left(x\;|\;\hat{T}\right)}-\sqrt{\hat{P}(x)}\right]}^{2}\;dx$$

on the predicted degree distribution as a function of c. As is visible, the error on the distribution decreases as c tends to its maximum value of N−1. For c=N−1, some residual uncertainty remains due to the uncertainty on the neural network parameters θ.

In Fig. 6b and c, we show the expected Hellinger error (Eq. 18) on the maximum likelihood estimator $\hat{P}$ as a function of *σ*, for both the degree and triangle distributions, i.e. x∈{k,t}. In addition, we also show the behavior of the expected relative entropy


(19) $$E_{\hat{T}}\int P\left(x\;|\;\hat{T}\right)\log\;\left(\frac{P\left(x\;|\;\hat{T}\right)}{\hat{P}(x)}\right)dx$$

and the total SD


(20) $$s^{2}=\int E_{\hat{T}}{\left[P\left(x\;|\;\hat{T}\right)-\hat{P}(x)\right]}^{2}\;dx.$$

All three metrics reflect the noise on the training data, providing similarly behaved, meaningful uncertainty quantification. As the noise tends to 0, some residual uncertainty again remains, while for very high noise levels, the uncertainty begins to plateau. Our method thus manages to capture the uncertainty arising from both sources: the nonconvexity of J and the noise σ on the data.

## Discussion

In this work, we have demonstrated a performative method to estimate network adjacency matrices from time series data. We showed its effectiveness at correctly and reliably inferring networks in a variety of scenarios: convex and nonconvex cases, low- to high-noise regimes, and equations that are both linear and nonlinear in A. We were able to reliably infer power line failures in the national power grid of Great Britain, and the connectivity matrix of an economic system covering all of Greater London. We showed that our method is well able to handle inference of hundreds of thousands to a million edge weights, while simultaneously giving uncertainty quantification that meaningfully reflects both the nonconvexity of the loss function as well as the noise on the training data. Our method is significantly more accurate than MCMC sampling and outperforms OLS regression in all except the virtually noiseless and fully determined cases. This is an important improvement since large amounts of data are typically required to ensure the network inference problem is fully determined, which may often not be available, as suggested in the power grid study. Unlike regression, our method also naturally extends to the case of nonlinear dynamics. In conjunction with our previous work (31), we have now also demonstrated the viability of using neural networks for parameter calibration in both the low- and high-dimensional case. Our method is simple to implement as well as highly versatile, giving excellent results across a variety of problems. All experiments in this work were purposefully conducted on a standard laptop CPU, typically taking on the order of minutes to run.

Many lines for future research open up from this work. First, a thorough theoretical investigation of the method is warranted, establishing rigorous convergence guarantees and bounds on the error of the posterior estimate. Another direction is further reducing the amount of data required to accurately learn parameters, and in future research the authors aim to address the question of learning system properties from observations of a single particle trajectory at the mean-field limit (62, 63). In this work, we have also not considered the impact of the network topology on the prediction performance, rather focusing on the physical dynamics of the problem. An interesting question is to what degree different network structures themselves are amenable to or hinder the learning process.

Over the past decade much work has been conducted into graph neural architectures (64, 65), the use of which may further expand the capabilities of our method. More specialized architectures may prove advantageous for different (and possibly more difficult) inference tasks, though we conducted a limited number of experiments with alternatives (e.g. autoencoders, cf. Fig. S4) and were unable to find great performance improvements. Finally, one drawback of our proposed method in its current form is it that it requires differentiability of the model equations in the parameters to be learned; future research might aim to develop a variational approach to expand our method to weakly differentiable settings.

## Supplementary Material

pgae063_Supplementary_Data

## Data Availability

Code and synthetic data can be found under https://github.com/ThGaskin/NeuralABM. It is easily adaptable to new models and ideas. The code uses the utopya package (utopia-project.org, utopya.readthedocs.io/en/latest) (66, 67) to handle simulation configuration and efficiently read, write, analyze, and evaluate data. This means that the model can be run by modifying simple and intuitive configuration files, without touching code. Multiple training runs and parameter sweeps are automatically parallelized. The neural core is implemented using pytorch (pytorch.org). All datasets have been made available, together with the configuration files needed to reproduce the plots. Detailed instructions are provided in Supplementary material and the repository.

## Note

a
developers.google.com/maps/documentation/distance-matrix.

## Preprints

This manuscript was posted on a preprint: arxiv.org/abs/2303.18059.
