# Supplementary material for: Inferring networks from time series: A neural approach
Source: PNAS Nexus. 2024 Feb 9;3(4):pgae063. doi: 10.1093/pnasnexus/pgae063 (PMC10978060; doi:10.1093/pnasnexus/pgae063)
Supplement: pgae063_Supplementary_Data [file pgae063_supplementary_data.pdf]

Supporting Information

Neural networks

Notation and Terminology. A *neural network* is a sequence of length $L \geq 1$ of concatenated transformations. Each *layer* of the net consists of L_i *neurons*, connected through a sequence of *weight matrices* $\mathbf{W}_i \in \mathbb{R}^{L_{i+1} \times L_i}$. Each layer applies the transformation

$$\sigma_i(\mathbf{W}_i \mathbf{x} + \mathbf{b}_i)$$

to the input $\mathbf{x}$ from the previous layer, where $\mathbf{b}_i \in \mathbb{R}^{L_{i+1}}$ is the *bias* of the i-th layer. The function $\sigma_i : \mathbb{R}^{L_{i+1}} \to \mathbb{R}^{L_{i+1}}$ is the *activation function*; popular choices include the *rectified linear unit* (ReLU) activation function $\sigma(x) = \max(x, 0)$, and the *sigmoid activation function* $\sigma(x) = (1 + e^{-x})^{-1}$. A neural net has an *input layer*, an *output layer*, and *hidden layers*, which are the layers in between the in- and output layers. If a network only has one hidden layer, we call it *shallow*, else we call the neural net *deep*.

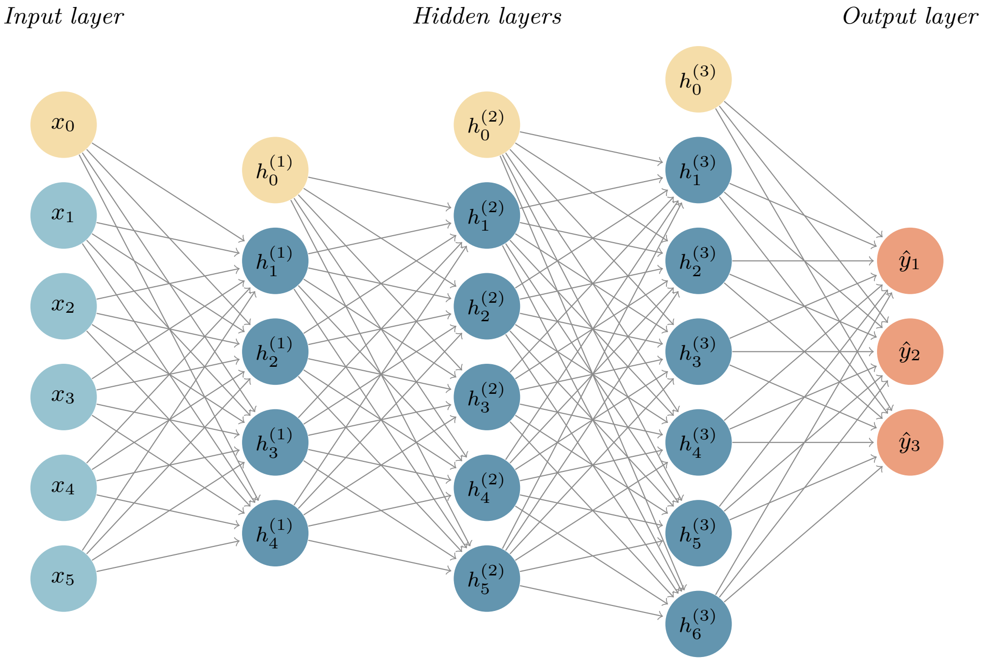

Fig. S1. Example of a deep neural network with 3 hidden layers. The inputs (light blue nodes) are passed through the layers, with links between layers representing the weight matrices $\mathbf{W}$. Each layer also applies a *bias* (yellow nodes), with the network finally producing an output (orange).

Choice of architecture. Here we provide additional studies to justify our choice of neural architecture. To select an appropriate architecture, we ran a hyperparameter sweep on synthetic Kuramoto data with $N = 100$ nodes, 70 training datasets and $L = 10$ time steps per dataset. Using a simple feed-forward architecture, we performed a sweep over the number of layers, nodes in each layer and activation functions used. Figures S2a—d show that using 5 layers with 20 nodes per layer reduces the training loss J optimally, and that further increasing the layer size only marginally reduces the L^1 prediction error. In addition, using very large, highly overparametrised models has the disadvantage of increased computational cost. Similar results hold for the Harris-Wilson dynamics eq. [6].

We also considered the use of different activation functions on the deep and final layers of the neural network (cf. fig. S3). On the inner layers, both a sigmoid and a hyperbolic tangent produce good results, while using anything other than the hard sigmoid on the final layer leads to poor results.

Lastly, using an autoencoder architecture instead of a feed-forward network produces no tangible benefits, so in the interest of simplicity we choose a simple feed-forward architecture S4.

Inferring line failures in the British power grid

Initialisation of the neural network weights. We initialise the neural network's weights with a prior $\pi^0(\boldsymbol{\theta})$ in such a way that the prior $\pi^0(\hat{\mathbf{A}})$ is a delta distribution on the complete graph, $\pi(\hat{a}_{ij}) \sim \delta(1)\ \forall i, j$. This can easily be achieved by training the neural network using the simple loss function

$$J = \|\hat{\mathbf{A}} - \mathbf{1}\|_2,$$

with $\mathbf{1} \in \mathbb{R}^{N \times N}$ a matrix of ones in all entries except on the diagonal, where it is zero. This only requires a few training steps, and helps maximise the sampling domain on each edge, making the calculations of p-values in our example more straightforward. However, we should stress that this initialisation is not necessary to obtain good calibration results.

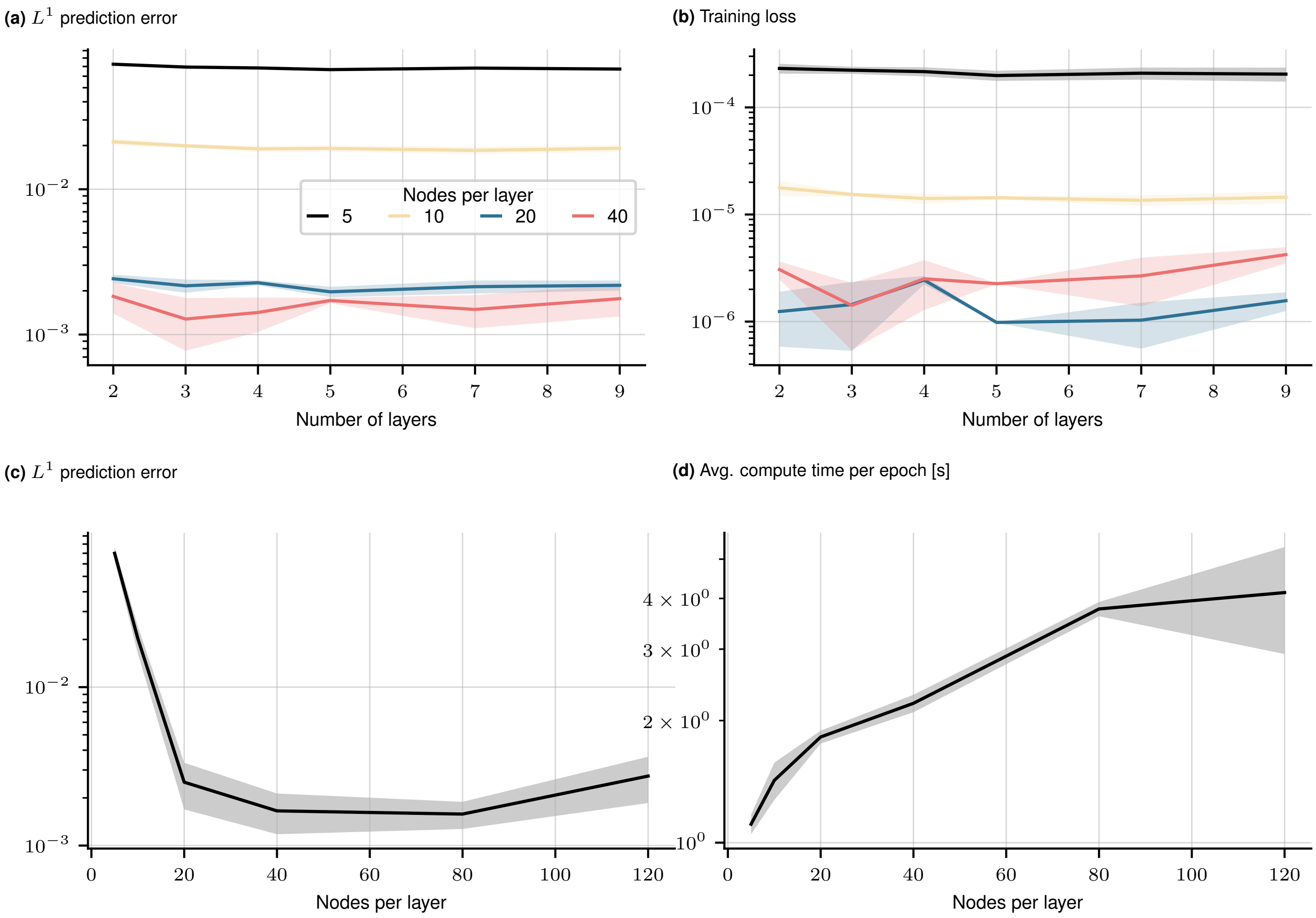

Fig. S2. Hyperparameter sweep results across neural network depth and width on synthetic Kuramoto data with $N = 100$. **(a)**: the total L^1 prediction error eq. [16] on the network adjacency matrix. **(b)**: the training loss J, both as a function of the number of layers and the number of nodes per layer in a simple feed-forward architecture. **(c)**: the L^1 prediction error as a function of the number of nodes per layer on a neural network with 5 layers. Increasing the layer size only marginally improves the prediction quality beyond a size of 20, and even decreases it for layer sizes greater than 80. **(d)**: average compute time per epoch as a function of the layer width.

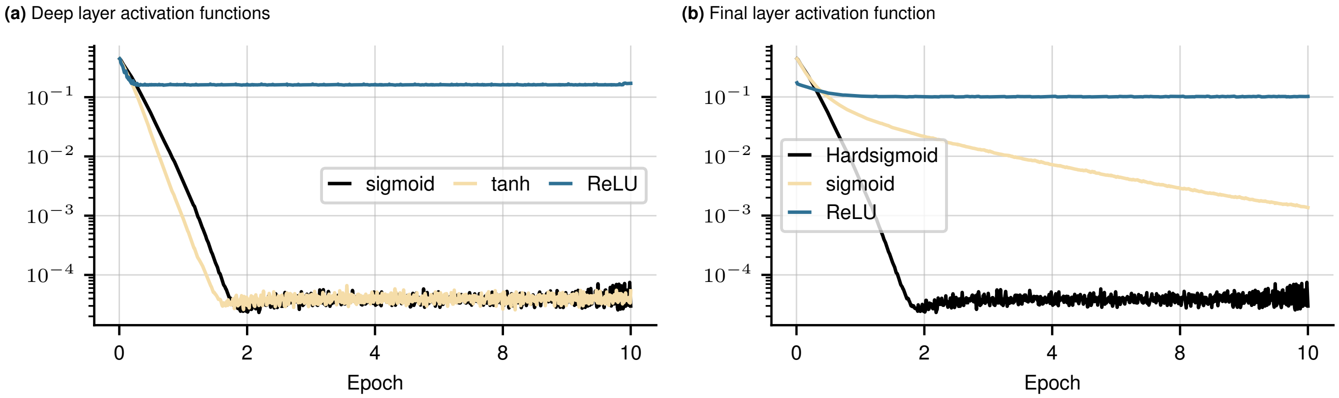

Fig. S3. Hyperparameter sweep results across neural network activation functions on the same data as in fig. S2. **(a)**: the total L^1 prediction error on the network adjacency matrix as a function of the activation functions used on the deep layers. On the final layer, the hard sigmoid is used. **(b)**: the L^1 prediction error as a function of the final layer activation function. On the inner layers, the hyperbolic tangent is used.

Constructing the admittance matrix. Here we provide some additional information on the calculation of the network edge weights from the data. The weights quantify the admittance (inverse impedance) of the line, that is, how easily the line can transmit electrical current. The characteristic impedance of a transmission line is given by

$$Z_0 = \sqrt{\frac{R + i\Omega L}{i\Omega C}}, \qquad [21]$$

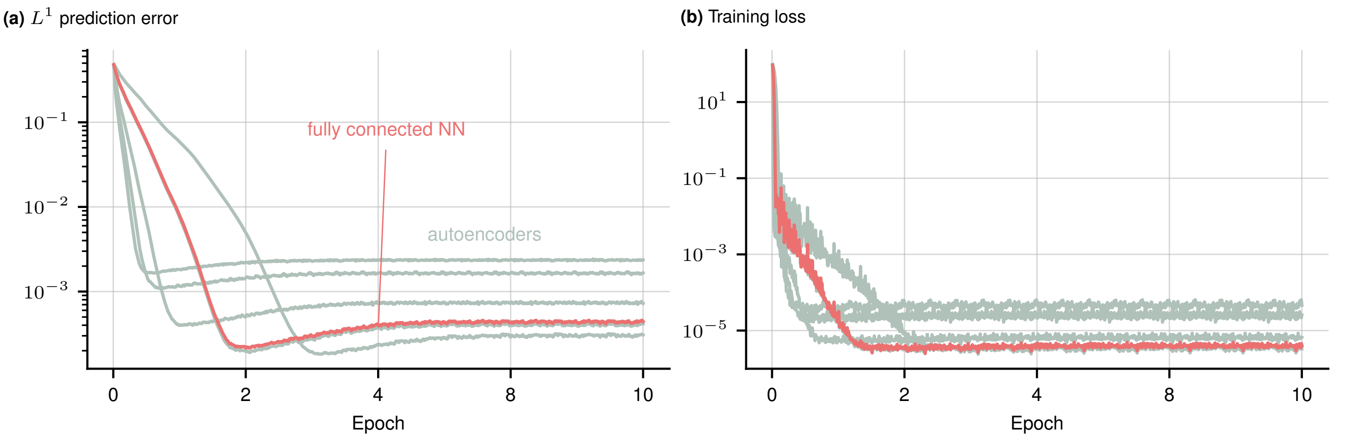

Fig. S4. L^1 prediction error **(a)** and total training loss **(b)** for various autoencoder architectures with different depths and widths (grey lines) and the simple feed-forward architecture (red) used in this work. The autoencoder architectures range from 25 neurons across 3 layers to about 430 neurons across 11 layers, and synthetic Kuramoto on a network with $N = 200$ nodes was used.

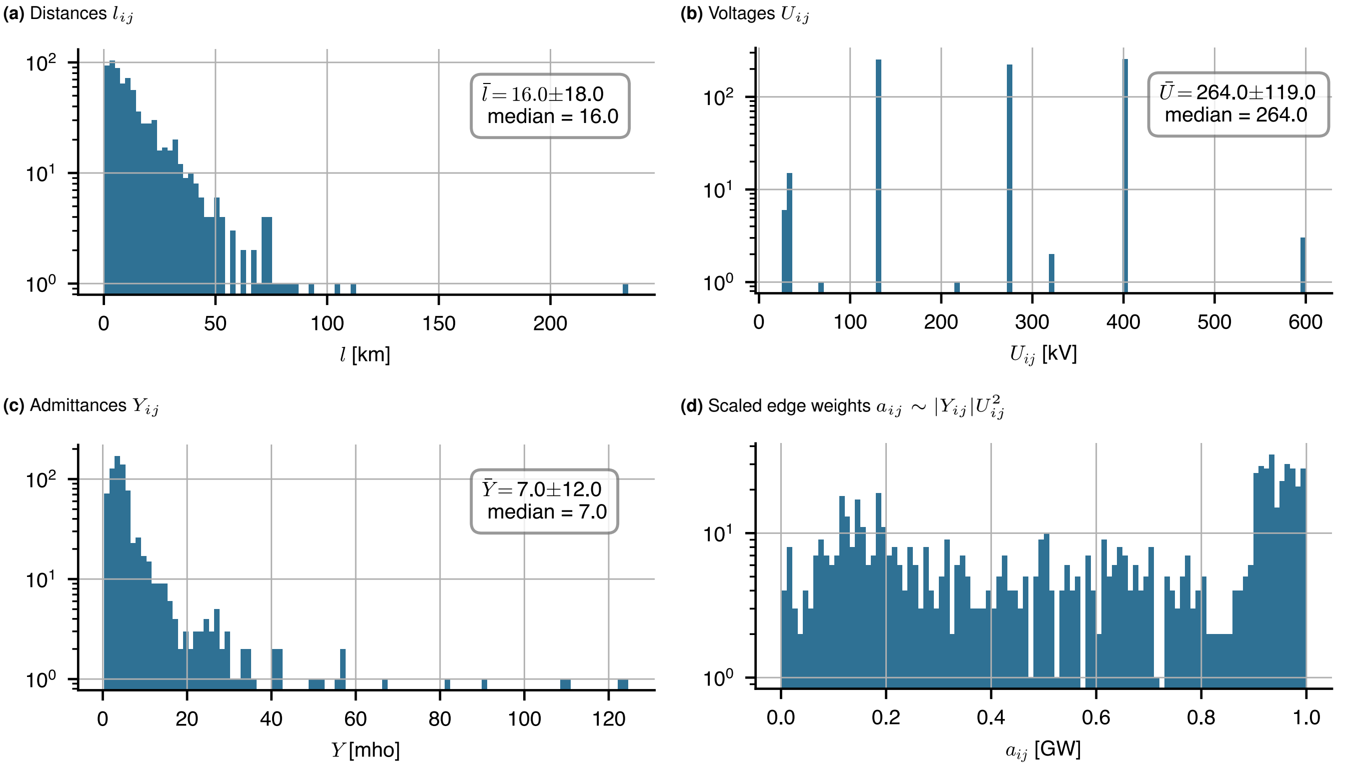

Fig. S5. Line data statistics for the British power grid. **(a)** Histogram of the node distances in the network, with mean, standard deviation, and median given. **(b)** Histogram of the line voltages. **(c)** Histogram of the line admittances. As is visible, a small number of short lines artifically skew the distribution. **(d)** The resulting normalised edge weights. Short edges with an artificially high admittance are assigned a random value in [0.9, 1].

with i the complex unit, $\Omega = 50$ Hz the grid frequency (67), L the cable inductance, and C its capacitance. We use the following values for a copper conductor with a cross-section of 1000 mm^2, provided by a standard manufacturer[§] of power grid cables: $R = 0.0276\ \Omega/\mathrm{km}$ (AC resistance), $L = 0.41$ mH/km, $C = 0.150\ \mu$F/km (assuming a single core). The total impedance along the line is then given by

$$Z(l) = Z_0 \sinh(\gamma l), \qquad [22]$$

where l is the length of the line, and the propagation constant γ is given by

$$\gamma = \sqrt{(R + i\Omega L)(i\Omega C)}. \qquad [23]$$

[§] caledonian-cables.co.uk/products/hv/400kv.shtml

In these calculations we neglect the conductance of the dielectric along the line, which is neglibile at the distances present in the network. The admittance of the line is then given by

$$Y_{ij} = Z_{ij}^{-1}, \quad [24]$$

and the total edge weight a_{ij} by

$$a_{ij} = |Y_{ij}|U_{ij}^2. \quad [25]$$

Long lines typically carry two to four times as many cables as shorter lines. We account for this by multiplying the admittance of lines over 10 km by a factor of 2, and those over 80 with a factor of 3 (admittances are additive). Since the neural network outputs values in $[0, 1]$, we scale the edge weights to this range. However, a small number of short lines artificially skew the distribution (see figure S5); these are mainly small segments designed to more accurately capture the geometry of a longer line, though sometimes they represent short lines to transformers, power stations, etc. To reduce their impact on the weight distribution, instead of dividing the weights by the maximum value, we divide by the mean and truncate all weights to $[0, 1]$:

$$a_{ij} \to \min(1, a_{ij}/\langle a_{ij}\rangle). \quad [26]$$

The scaling factor is absorbed into the coupling coefficient κ. Those edges with weight exactly equal to 1 are reassigned a value chosen uniformly at random in $[0.9, 1.0]$. As is visible in fig. S5d, the resulting distribution of the weights a_{ij} is more uniform than that of the distances l_{ij} and admittances Y_{ij}, since longer lines' lower admittance is often compensated by a higher voltage.

These calculations do not account for, among other things, the fact that we are connecting the nodes with straight lines rather than the real line trajectory, are not discerning between overground and underground lines, are assuming a single core per cable, and are not considering differences in transmission line heights, geometries, and materials. These inaccuracies are absorbed into the coefficients α and β, which we tune manually in order to allow for stable phase-locking.

Comparative performance analysis

In figure S6 we show the equivalent of fig. 5a for the case of second-order Kuramoto dynamics (eq. [3] with $\alpha = 1$). We again see the neural scheme outperforming OLS regression at very low levels of the noise, though the performance improvement is less stark than in the first-order case. MALA marginally outperforms the neural scheme at very high noise levels. Enough data is used to ensure full invertibility of the Gram matrix ($\mathfrak{c} = N - 1$).

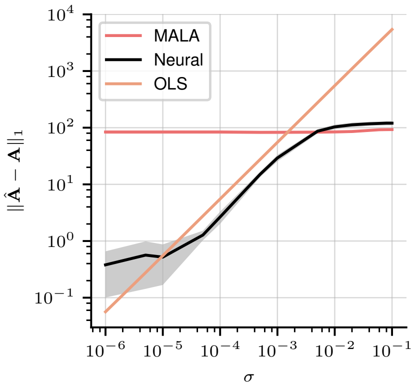

Fig. S6. L^1 prediction error of the neural scheme, the preconditioned Metropolis-adjusted Langevin sampler, and OLS regression as a function of the noise variance σ on the training data, for second-order Kuramoto dynamics ($\alpha = 1$).

Details on the code

The code is uploaded to the Github repository as given in the main text. The two models relevant to this work are `Kuramoto` and `HarrisWilsonNW`.

Installation. Detailed installation instructions are given in the repository. First, clone the repository, install the `utopya` package and all the required additional components into a virtual environment, for example via `PyPi`. In particular, install `pytorch`. Enter the virtual environment. Then, from within the project folder, register the project:

```
utopya projects register .
```

You should get a positive response from the utopya CLI and your project should appear in the project list when calling:

```
utopya projects ls
```

Note that any changes to the project info file need to be communicated to utopya by calling the registration command anew. You will then have to additionally pass the `-exists-action overwrite` flag, because a project of that name already exists. See

```
utopya projects register --help
```

for more information. Finally, register the project and its models via

```
utopya projects register . --with-models
```

Running the code. To run a model, execute the following command:

```
utopya run <model_name>
```

By default, this runs the model with the settings in the <model_name>_cfg.yml file. All data and the plots are written to an output directory, typically located in `~/utopya_output`. To run the model with different settings, create a `run_cfg.yml` file and pass it to the model like this:

```
utopya run <model_name> path/to/run_cfg.yml
```

This is recommended rather than changing the default settings, because the defaults are parameters that are known to work and you may wish to fall back on in the future.

Plots are generated using the plots specified in the <model_name>_plots.yml file. These too can be updated by creating a custom plot configuration, and running the model like this:

```
utopya run <model_name> path/to/run_cfg.yml --plots-cfg path/to/plot_cfg.yml
```

See the Utopia tutorial for more detailed instructions.

All the images in this article can be generated using so-called *configuration sets*, which are complete bundles of both run configurations and evaluation configurations. For example, to generate the predictions on the random network with $N = 1000$ nodes (fig. 5d–e) for the Kuramoto model, you can call

```
utopya run Kuramoto --cfg-set N_1000_example
```

This will run and evaluate the `Kuramoto` model with all the settings from the `Kuramoto/cfgs/N_100_example/run.yml` and `eval.yml` configurations.

Parameter sweeps. Parameter sweeps are automatically parallelised by `utopya`, meaning simulation runs are always run concurrently whenever possible. The data is automatically stored and loaded into a data tree. To run a sweep, simply add a `!sweep` tag to the parameters you wish to sweep over, and specify the values, along with a default value to be used if no sweep is performed:

```
param: !sweep
  default: 0
  values: [0, 1, 2, 3]
```

Then in the run configuration, add the following entry:

```
perform_sweep: true
```

Alternatively, call the model with the flag `--run-mode sweep`. The configuration sets used in this work automatically run sweeps whenever needed, so no adjustment is needed to recreate the plots used in this work.

Initialising the neural net. The neural net is controlled from the `NeuralNet` entry of the configuration:

```
NeuralNet:
  num_layers: 4
  nodes_per_layer:
    default: 20
    layer_specific:
      1: 10
      2: 15
  biases:                 # optional; if this entry is omitted no biases are used
    default: ~            # default is None (indicted by a tilde in YAML)
    layer_specific:
      0: default          # use pytorch default (xavier uniform)
      -1: [-1, 1]         # uniform initialisation on a custom interval
  activation_funcs:
    default: sigmoid
    layer_specific:       # optional
      1: tanh
      3:
        name: HardTanh   # you can also pass a function that takes additional args and/or kwargs
        args:
          - -2  # min_value
          - +2  # max_value
  learning_rate: 0.001    # optional; default is 0.001
  optimizer: SGD          # optional; default is Adam
```

`num_layers` specifies the depth of the net; `nodes_per_layer` controls the architecture: provide a `default` size, and optionally any deviations from the default under `layer_specific`. The keys of the `layer_specific` entry should be indices of the layer in question. The optional `biases` entry determines whether or not biases are to be included in the architecture, and if so how to initialise them. A default and layer-specific values can again be passed. Setting an entry to `default` initialises the values using the pytorch default initialiser, a Xavier uniform initialisation. Passing a custom interval instead initialises the biases uniformly at random on that interval, and passing a tilde `~` (`None` in YAML) turns the bias off. `activation_funcs` is a dictionary specifying the activation functions on each layer, following the same logic as above. Any pytorch activation function is permissible. If a function requires additional arguments, these can be passed as in the example above. Lastly, the `optimizer` keyword takes any argument allowed in pytorch. The default optimizer is the Adam optimizer (39) with a learning rate of 0.02.

The neural net can be initialised from different initial values in the parameter space by changing the *random seed* in the configuration:

```
seed: 42
```

Sweeping over different initialisations is achieved by sweeping over the seed, as described in the previous section.

Training the neural net. The `Training` entry of the configuration controls the training process:

```
Training:
  batch_size: 2
  device: cpu
  true_parameters:
    sigma: 0.0
  loss_function:
    name: MSEloss
    # can pass additional args and kwargs here ...
```

You must specify the noise level to use for the ABM during training; the default value is 0. Under the `loss_function` key you can specify the loss function to use, and pass any arguments or keyword arguments it may require using an `args` or `kwargs` key. You can use any available pytorch loss function.

The `device` key sets the training device. The default is the CPU, but you can also train on the GPU by setting the device to `cuda`. Note that on Apple Silicon, the device name is `mps`. Make sure you have installed the correct package for your device, and note that, as of writing, some pytorch functions required for our code (e.g., the trace and hard sigmoid functions) had not yet been implemented for MPS, hence GPU training on Apple Silicon devices was not possible.

Kuramoto model

Neural Network Architecture. The following is the default configuration for the neural network and training settings used for the Kuramoto model:

```
Kuramoto:
  NeuralNet:
    num_layers: 5
    nodes_per_layer:
      default: 20
    biases:
      default: ~   # No biases
    activation_funcs:
      default: tanh
      layer_specific:
        -1: HardSigmoid   # hard sigmoid on the last layer
    learning_rate: 0.002
    optimizer: Adam
  Training:
    batch_size: 2
    loss_function:
      name: MSELoss
      kwargs:
        reduction: sum
    true_parameters:
      sigma: 0
```

Training. We rewrite [3] as a vector-matrix equation,

$$\alpha \frac{\mathrm{d}^2 \boldsymbol{\varphi}}{\mathrm{d}t^2} + \beta \frac{\mathrm{d}\boldsymbol{\varphi}}{\mathrm{d}t} = \mathbf{P} + \kappa \mathrm{diag}(\mathbf{A}\boldsymbol{\Gamma}(\boldsymbol{\varphi})),$$

which is more amenable to machine learning purposes, since it can make use of fast matrix-multiplication operations. $\boldsymbol{\Gamma}$ is the interaction kernel matrix, $\Gamma_{ij} = \sin(\varphi_j - \varphi_i)$. We the train the model using the following numerical operation: let $\boldsymbol{\varphi}(t) = (\varphi_1(t), ...\varphi_N(t))$ be the current phases of the N nodes, $\dot{\boldsymbol{\varphi}}(t) = (\varphi_1(t) - \varphi_1(t-1), ..., \varphi_N(t) - \varphi_N(t-1)$ the vector of phase derivatives, $\boldsymbol{\omega} = (\omega_1, ..., \omega_N)$ the vector of eigenfrequencies, and $\boldsymbol{\Gamma}(t) = (\sin(\varphi_j(t) - \varphi_i(t))_{ij}$; then in each iteration of the first-order Kuramoto model ($\beta \neq 0$), we do

$$\boldsymbol{\varphi}(t+1) = \boldsymbol{\varphi}(t) + \frac{1}{\beta}\left(\boldsymbol{\omega}(t) + \mathrm{diag}\left(\hat{\mathbf{A}}\boldsymbol{\Gamma}(t)\right)\right)\mathrm{d}t, \qquad [27]$$

where diag(·) takes the diagonal elements of the matrix. For the second-order model ($\alpha \neq 0$), we do

$$\boldsymbol{\varphi}(t+1) = \boldsymbol{\varphi}(t) + \frac{1}{\alpha}\left[\left(\boldsymbol{\omega}(t) + \mathrm{diag}\left(\hat{\mathbf{A}}\boldsymbol{\Gamma}(t)\right) - \beta\dot{\boldsymbol{\varphi}}(t)\right)\mathrm{d}t + \dot{\boldsymbol{\varphi}}(t)\right]\mathrm{d}t. \qquad [28]$$

In the first-order case, the initial conditions required are $\boldsymbol{\varphi}(0)$, in the second order case, we need $\boldsymbol{\varphi}(0)$ and $\boldsymbol{\varphi}(1)$ (i.e. the initial phases and initial velocities).

Running the code. Configuration sets to reproduce all numerical experiments except the British power grid inference are provided; for instance, to produce the predictions for a random network with $N = 100$ nodes, simply run

```
utopya run Kuramoto --cfg-set N_1000_example
```

Harris-Wilson model

Neural Network Architecture. The following is the default configuration for the neural network and training settings used for the Harris-Wilson model:

```
HarrisWilsonNW:
  NeuralNet:
    num_layers: 2
    nodes_per_layer:
      default: 20
    biases:
      default: ~   # No biases
    activation_funcs:
      default: tanh
```

```
    layer_specific:
      -1: sigmoid   # sigmoid on the last layer
  learning_rate: 0.002
  optimizer: Adam
Training:
  batch_size: 2
  loss_function:
    name: MSELoss
    kwargs:
      reduction: sum
  true_parameters:
    sigma: 0
```

Training. We use the following matrix form of the Harris-Wilson equations to train the neural net. Let $\mathbf{D} \in \mathbb{R}^M$, $\mathbf{O} \in \mathbb{R}^N$, $\mathbf{W} \in \mathbb{R}^M$ be the demand vector, origin zone size vector, and destination zone size vector respectively. The the dynamics are given by

$$\mathbf{D} = \mathbf{W}^\alpha \odot \left[\left(\mathbf{C}^\beta \right)^\top (\mathbf{O} \odot \mathbf{Z}) \right] \in \mathbb{R}^M, \qquad [29]$$

with $\odot$ indicating the Hadamard product, and elementwise exponentiation. $\mathbf{Z} \in \mathbb{R}^N$ is the vector of normalisation constants

$$\mathbf{Z}^{-1} = \mathbf{C}^\beta \mathbf{W}^\alpha. \qquad [30]$$

The dynamics then read

$$\dot{\mathbf{W}} = \mathbf{W} \odot \epsilon(\mathbf{D} - \kappa \mathbf{W}) \qquad [31]$$

with given initial conditions $\mathbf{W}(t = 0) = \mathbf{W}_0$. This formulation is more conducive to machine learning purposes, since it contains easily differentiable matrix operations and does not use for-loop iteration.

Running the code. This is analogous to the Kuramoto case. To reproduce the plot from the main article, run the following command:

```
utopya run HarrisWilsonNW --cfg-set London_dataset
```
